# Clinical results of proton beam therapy for advanced neuroblastoma

**DOI:** 10.1186/1748-717X-8-142

**Published:** 2013-06-12

**Authors:** Yoshiko Oshiro, Masashi Mizumoto, Toshiyuki Okumura, Shinji Sugahara, Takashi Fukushima, Hitoshi Ishikawa, Tomohei Nakao, Takayuki Hashimoto, Koji Tsuboi, Haruo Ohkawa, Michio Kaneko, Hideyuki Sakurai

**Affiliations:** 1 Department of Radiation Oncology, University of Tsukuba, Tennodai 1-1-1, Tsukuba, Ibaraki, Japan; 2 Department of Pediatrics, University of Tsukuba, Ibaraki, Japan; 3 Department of Pediatric Surgery, University of Tsukuba, Ibaraki, Japan; 4 Department of Radiation Oncology, Tokyo Medical University Ibaraki Medical Center, Ami, Inashiki, Ibaraki, Japan

**Keywords:** Neuroblastoma, Proton therapy, Radiotherapy, Late toxicity, Pediatrics

## Abstract

**Purpose:**

To evaluate the efficacy of proton beam therapy (PBT) for pediatric patients with advanced neuroblastoma.

**Methods:**

PBT was conducted at 21 sites in 14 patients with neuroblastoma from 1984 to 2010. Most patients were difficult to treat with photon radiotherapy. Two and 6 patients were classified into stages 3 and 4, respectively, and 6 patients had recurrent disease. Seven of the 8 patients who received PBT as the initial treatment were classified as the high risk group. Twelve patients had gross residual disease before PBT and 2 had undergone intraoperative radiotherapy before PBT. Five patients received PBT for multiple sites, including remote metastases. Photon radiotherapy was used in combination with PBT for 3 patients. The PBT doses ranged from 19.8 to 45.5 GyE (median: 30.6 GyE).

**Results:**

Seven patients are alive with no evidence of disease, 1 is alive with disease progression, and 6 died due to the tumor. Recurrence in the treatment field was not observed and the 3-year locoregional control rate was 82%. Severe acute radiotoxicity was not observed, but 1 patient had narrowing of the aorta and asymptomatic vertebral compression fracture at 28 years after PBT, and hair loss was prolonged in one patient.

**Conclusion:**

PBT may be a better alternative to photon radiotherapy for children with advanced neuroblastoma, and may be conducted safely for patients with neuroblastoma that is difficult to manage using photon beams.

## Introduction

Neuroblastoma is the most common extracranial solid tumor in children. Half of newly diagnosed patients present with high risk disease that is widely metastatic and has large and invasive lesions in the advanced stage. Aggressive treatment is conducted in these cases because they are highly sensitive to radiotherapy and chemotherapy. However, despite recent progress with systemic therapy, the treatment outcome in high risk neuroblastoma is poor [1-5]. Advanced neuroblastoma also often recurs and the prognosis after recurrence is extremely poor [6-8], with Garaventa et al. finding survival rates of only 6.6% and 1.5% in patients with progression and relapse disease [7]. Recently, the superiority of the dose distribution in proton beam therapy (PBT) has been shown compared to the photon dose distribution especially in children [9-13]. However, to our knowledge, there are few reports on clinical outcomes after PBT. [14] We have mainly treated patients with advanced high risk neuroblastoma or recurrent disease using PBT since 1984. Herein, we report a retrospective review of the outcome and toxicity in these patients.

## Methods

### Patients

Fourteen patients with neuroblastoma received PBT at 21 sites from 1984 to 2010 at our institute. The patient characteristics are shown in Table 1. The patients were 6 boys and 8 girls with a median age of 3 years old (range 1 to 6 years old). PBT was conducted because photon beam radiotherapy was difficult due to a large irradiation area involving normal organs such as the liver, heart, and gastrointestinal tract for 8 patients (Nos. 1–3, 6, 7, 12–14), and because PBT was considered a better option for reduction of the dose to the eyes for 4 patients with orbital, paranasal sinus and skull base disease (Nos. 4, 8, 9, 11). The other 2 patients (Nos. 5, 10) received PBT because of the wishes of their families. All patients had received chemotherapy before PBT.[2] Eight patients received PBT as initial treatment, including two with Stage 3 and six with Stage 4 disease classified by the International Neuroblastoma Staging System (INSS). The other 6 patients received PBT for recurrent disease. Twelve patients of the 14 had gross residual disease. Eleven patients were classified as high risk (i.e. those with stage 4 disease aged older than 1 year at diagnosis or those with stage 3 MYCN-amplified tumors), 2 with recurrent disease were intermediate risk (i.e. stage 3 disease aged older than 1 year with favorable histology and MYCN-non-amplified tumors), and insufficient biological and histological data were available to determine the risk group in 1 patient diagnosed in 1982 according to the international neuroblastoma risk group (INRG) staging system[15]. The treatment site was the abdomen and pelvis in 9 cases, head and neck in 7, thorax in 2, paravertebra in 1, acetabulum in 1, and skull in 1. Five patients received PBT at multiple sites for primary and metastatic lesions. Photon radiotherapy was used in combination with PBT for 3 patients for lymph nodes and distant metastases. Intraoperative radiotherapy (IORT) had undergone for the recurrent disease in 2 patients.

**Table 1:** Background of 14 patients treated with proton beam therapy

**Patient No**	**Age at PBT**	**Risk**	**INSS stage**	**Site**	**Surgery**	**Gross residual disease at PBT**	**PBT dose (GyE)**	**Port No.**	**Sedation**	**Respiratory gating**	**X-ray**
1	1	Unknown	3	upper abdomen	open biopsy	Yes	28.6	1	yes	no	none
2	2	High	4	retroperitoneum	complete resection	No	19.8	1	yes	yes	Photon for femoral bone
3	6	High	3	mediastinum	None	Yes	30.6	3	no	yes	Electron for axillary LN and skull
4	5	High	3	Paranasal sinus	None	No	19.8	2	no	no	Photon for whole neck LN
5	3	High	4	paravertebra	partial removal	Yes	39.6	1	no	yes	none
6	3	High	4	retroperitoneum	partial removal	Yes	30.6	2	no	yes	none
7	5	High	4	retroperitoneum	partial removal	Yes	30.6	2	no	yes	none
				skull base	None	No	19.8	2		no	
8	2	High	4	retroperitoneum	partial removal	No	19.8	2	yes	yes	none
				skull	None	Yes	19.8	1		no	
				orbit	None	Yes	19.8	1		no	
9	3	High	R^*^	orbit	none	Yes	43.7	1	yes	no	Cobalt for neck LN
10	6	High	R	paravertebra	None	Yes	45.5	2	no	no	none
				mediastinal node	None	Yes	45.5	2		yes	
11	6	High	R	skull base	None	Yes	33.7	2	no	no	none
				occipital bone	None	Yes	33.7	2		no	
12	2	Intermediate	R	retroperitoneum	none	Yes	41.4	2	yes	yes	Post IORT 12Gy
13	2	High	R	retroperitoneum	None	Yes	41.4	2	yes	yes	Post IORT 12Gy
				paraaorta LN^†^	None	Yes	30.6	2		yes	none
14	6	Intermediate	R	supraclavicular LN	None	Yes	30.6	1	no	no	
				acetabulum	None	Yes	30.6	2		no	

*R: recurrent disease, ^†^LN: lymph nodes.

### Proton therapy

Before treatment, CT images for PBT planning were obtained at intervals of 2–5 mm in the treatment position. The interval was determined based on the patient’s age, height and treatment site. For 10 patients with pelvic and thoracic disease, the CT image was obtained during the end expiratory phase using a respiratory gating system, as described previously [16,17]. The gross tumor volume (GTV) was defined as the tumor volume after remission induction chemotherapy for a primary tumor and the tumor volume before PBT for a recurrent tumor. The clinical target volume (CTV) was defined as the GTV plus a 1.5-cm margin and the PTV was defined as the CTV plus a 0.5- to 0.7-cm margin, in principle; however, the balance between toxicity and treatment effect was also taken into account in determining the CTV. Sedatives were administered for 5 patients aged 1 to 3 for planning CT and treatment.

Between 1994 and 2000, PBT was limited to 4 hours a day and 120 days a year according to proton beam availability from the National Laboratory for High Energy Physics. Beam lines were also limited to fixed vertical and horizontal beam lines, and patients were immobilized by Styrofoam box manually-hollowed out for individuals. From September 2001, the new hospital-based facility which includes rotational gantries, releases adequate energy proton beams from any direction, using the respiratory gating for 10 patients with pelvic and thoracic disease with the body immobilized using an individually shaped body cast (ESFORM; Engineering System Co., Matsumoto). Patients with head and neck tumors were also immobilized using individually manufactured thermoplastic masks. The treatment is provided 5 days in a week. Respiratory gating was used for the 10 patients with pelvic and thoracic disease. The photon equivalent dose (GyE) was defined as the physical dose (Gy) × the relative biological effectiveness of the proton beam assigned a value of 1.1. Before each treatment, correct placement of the patient relative to the radiation field was confirmed fluoroscopically. The given doses ranged from 19.8 to 45.5 GyE (median: 30.6 GyE) in 11 to 23 fractions. In Japan, the common dose for neuroblastoma with a complete response after chemotherapy is 19.8 Gy, and 10.8 Gy was added for grossly residual disease. In this series, higher doses were administered for patients with recurrent and chemotherapy-resistant disease. Patients underwent a routine physical examination once a week during PBT. After completion of PBT, patients were followed in combination with a pediatrician using CT, MRI, ^99m^Tc bone scans, and ^131^I- and ^123^I- metaiodobenzylguanidine scintigrams.

### Statistical analysis

Locoregional failure was defined as tumor progression in the anatomic compartment that contained the primary tumor (pelvis, abdomen, thorax, neck). The locoregional control rate was calculated from the start of PBT to the date of local failure in the irradiation field, marginal recurrence, or most recent local progression-free follow up. Statistical analyses were performed using SPSS software (SPSS Inc., Chicago, IL, USA). Acute and late toxicities associated with treatments were evaluated using the National Cancer Institute Common Toxicity Criteria for Adverse Events (CTCAE) version 4.0.

## Results

The results for the 14 patients are shown in Table 2. The median follow-up periods from diagnosis and the start of PBT were 40 months (M) (range: 17 M-30 years (Y)) and 21 M (5 M-29 Y), respectively, for all patients, and 46 M (25 M-30 Y) and 30 M (18 M-29 Y), respectively, for surviving patients. The planned irradiation was completed in all patients. At the time of analysis in 2012, 8 patients were alive. Of the 8 patients who received PBT as initial treatment, 6 (75%) were alive with no evidence of disease, 1 was alive with distant metastasis, and 1 had died from tumor progression. Of the 6 patients who received PBT for recurrence, 1 was alive with no evidence of disease and 5 had died from tumor progression. The initial progression sites were bone (n = 2), bone marrow (n = 2), liver (n = 1), brain (n = 1), and lymph nodes (n = 1).

**Table 2:** Clinical outcomes in 14 patients treated with proton beam therapy

**Patient No**	**Cause of death**	**Survival after PBT (M)**	**Progression**	Acute toxicity^*^	Late toxicity*
1	alive	349	none	none	vertebral growth retardation, narrowed aorta
2	alive	61	none	none	none
3	alive	40	none	none	none
4	alive	39	none	temporary hair loss, G1 pharyngitis	None
5	tumor	9	bone	G1 skin reaction	None
6	alive	20	none	None	G1 skin pigmentation
7	alive	20	bone marrow	None	none
8	alive	18	none	hair loss	thin hair
9	tumor	11	brain	G1 skin reaction	none
10	tumor	11	bone	none	none
11	tumor	31	bone marrow	none	none
12	tumor	27	lymph node	none	none
13	tumor	5	liver	none	-
14	alive	22	none	none	none

*G1: Grade 1 according to Criteria for Adverse Events version 3.0 of the National Cancer Institute and the late radiation morbidity scoring scheme of the Radiation Therapy Oncology Study Group/European Organization for Research and Treatment of Cancer.

**Figure 1:**
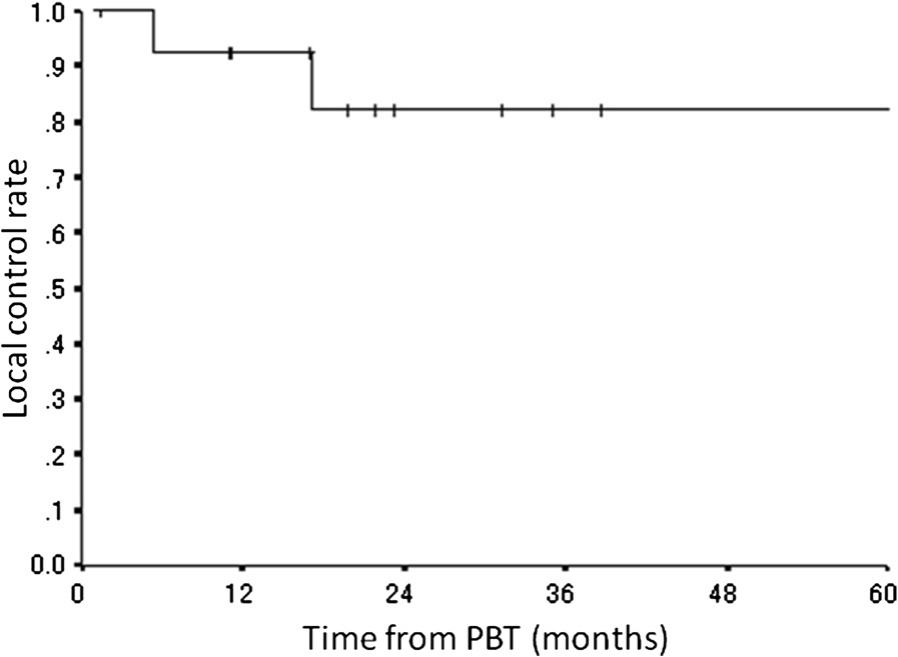
Local control rate for all patients.

**Figure 2:**
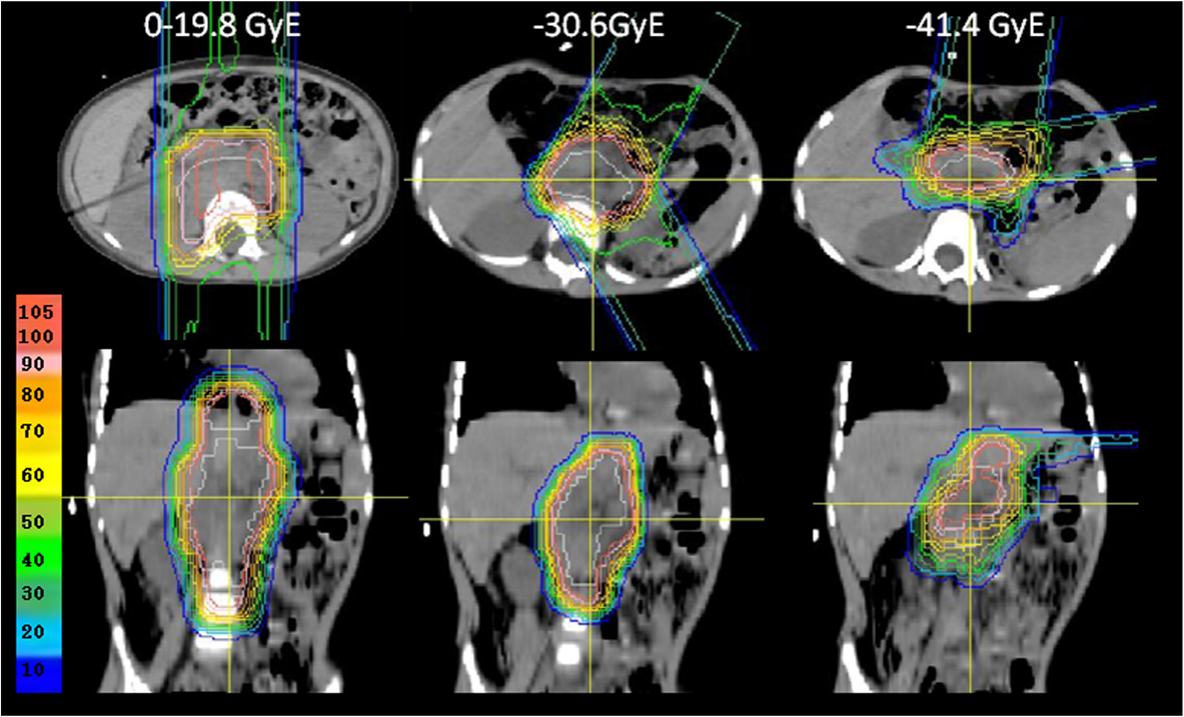
Dose distribution for patient No.12.

**Figure 3:**
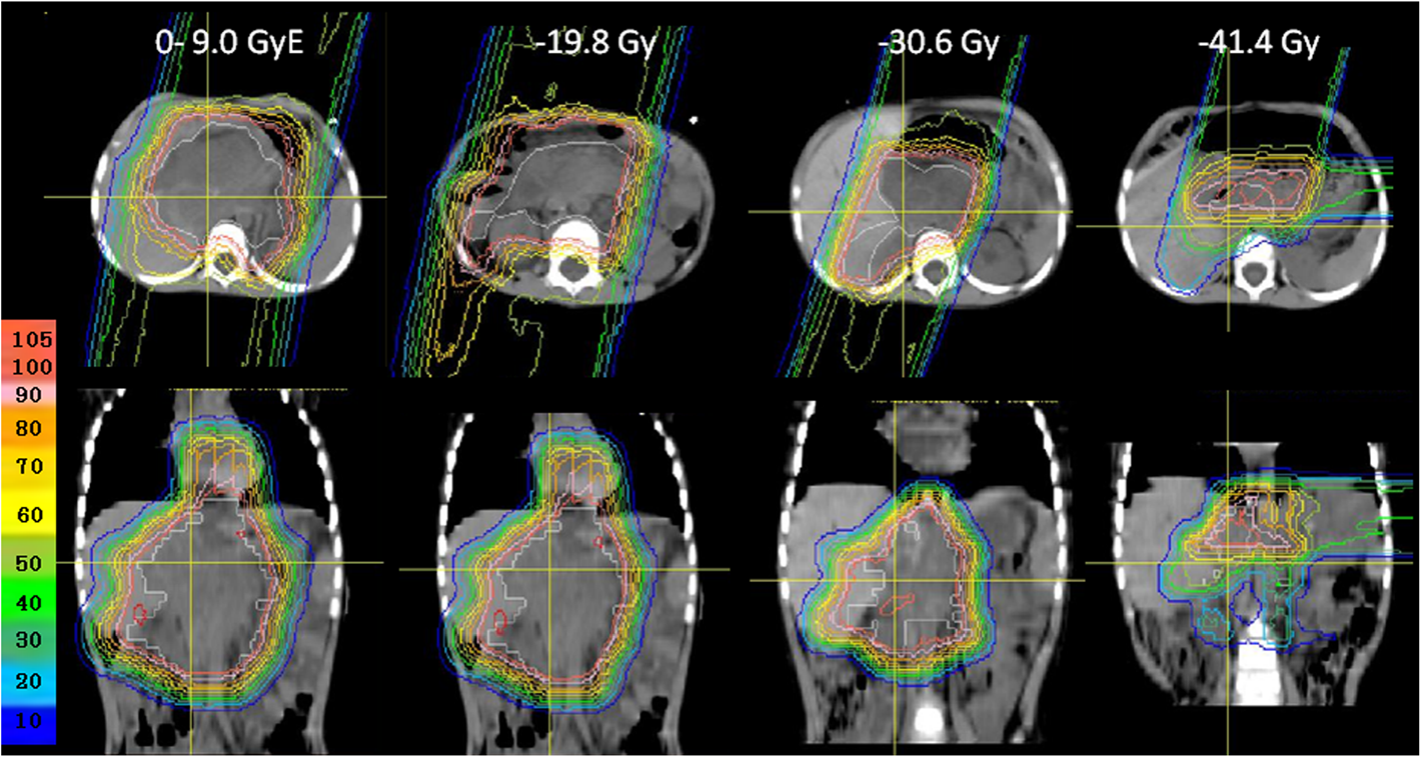
Dose distribution for patient No.13.

Recurrence in the treatment field was not observed, but marginal failures occurred in 2 patients (Nos. 12, 13), resulting in a 3-year a locoregional control rate of 82% (CI: 59-100%) (Figure 1). One patient (No. 12) received PBT for a bulky recurrent tumor. This patient received PBT of 19.8 GyE for the pre-chemotherapy tumor volume, 30.6 GyE for the gross tumor volume, and 41.4 GyE for the gross tumor volume excluding the IORT field (Figure 2). The PTV margins were 5 mm because of the large irradiation volume. After completion of PBT, the tumor had disappeared, but paraaortic lymph node metastases appeared below the first irradiation field 17 months after PBT. This recurrent lesion was irradiated with about a 40% dose of 19.8 GyE. The second patient (No. 13) also received PBT for a bulky recurrent tumor invading the hepatic portal region (Figure 3). The irradiation field was too large to add the margin of 1.5 cm, and the whole tumor was included in the treatment field with a margin of 7 mm for the dose of 30.6 GyE, but the left kidney was blocked after 10.8 GyE. The tumor at the Morison fossa was excluded from the treatment field after 30.6 GyE by considering tolerance of the liver, and the treatment field was reduced to the main retroperitoneal tumor. The tumor shrunk in size, but disease progression occurred in the portal region beyond the irradiation field 5 months after PBT.

**Figure 4:**
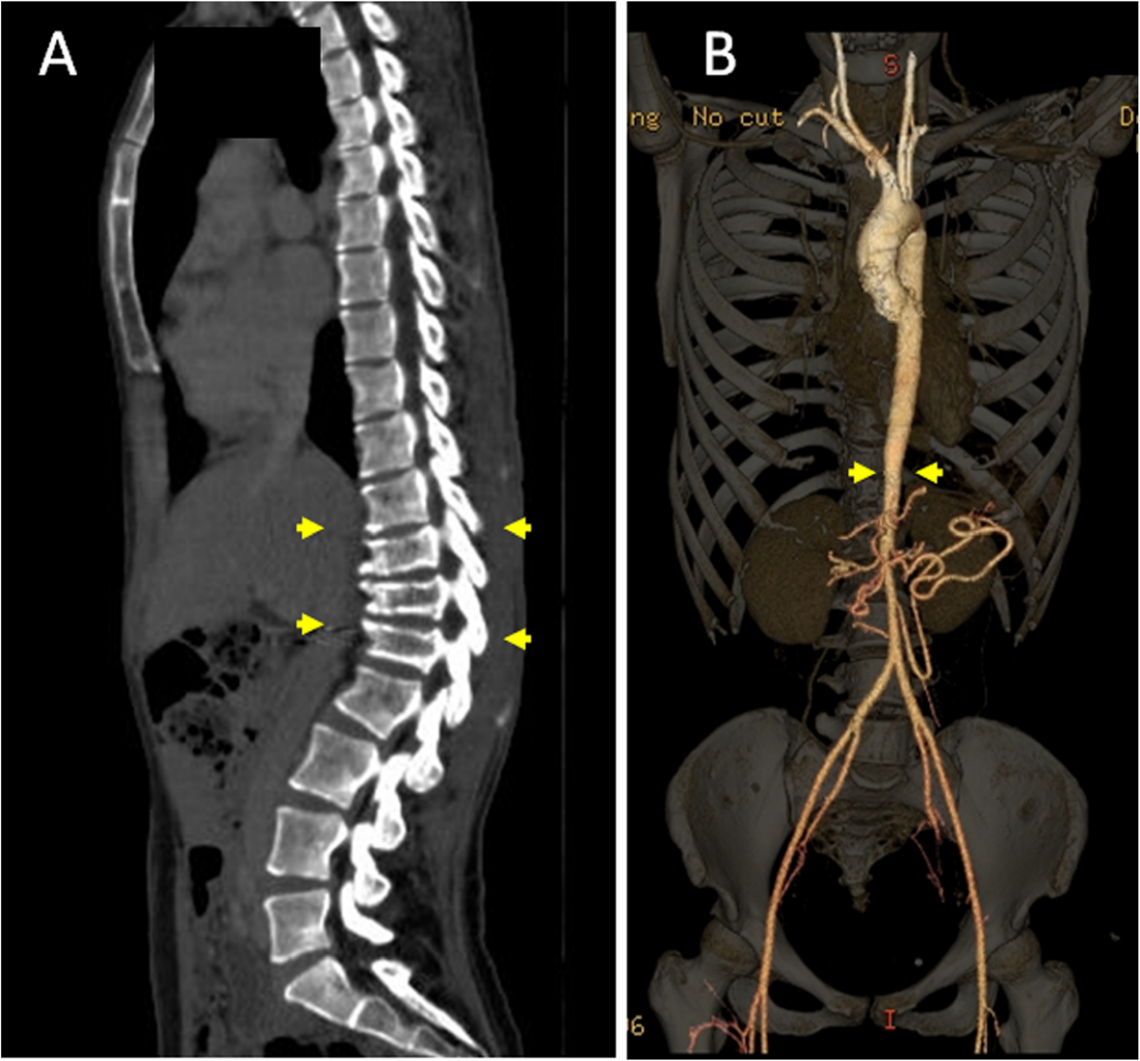
**CT scan 28 years after the PBT.** (**A**) Growth retardation in the ventral vertebral body. (**B**) Narrowing of the aorta below the irradiated field.

**Figure 5:**
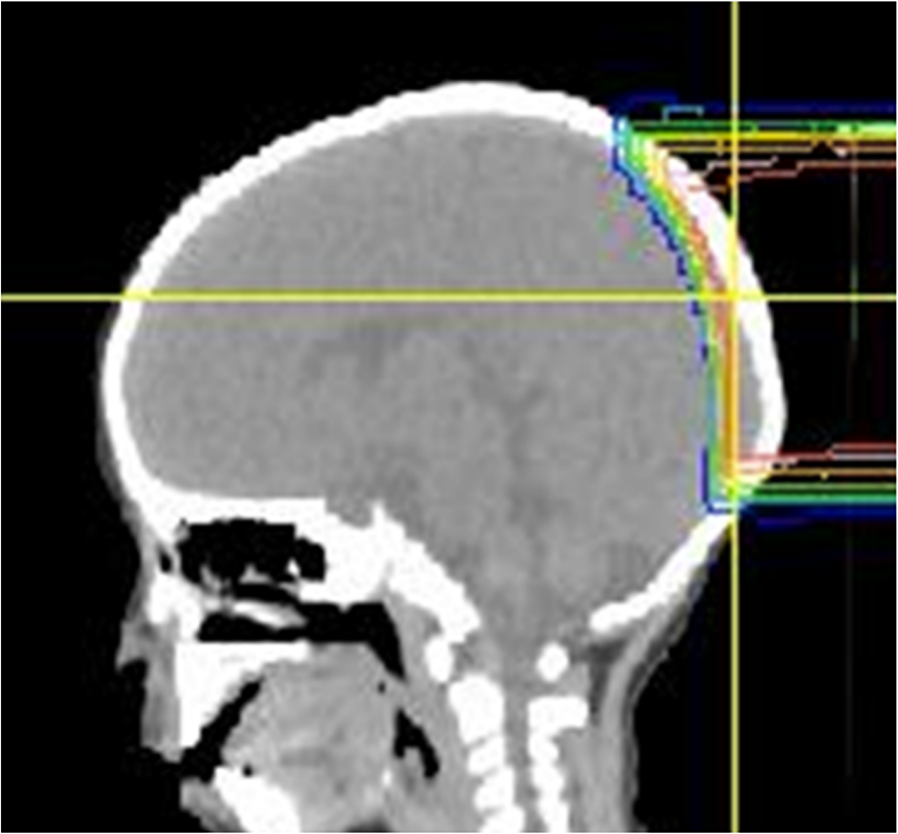
Proton irradiation of the skull reduces the dose to brain tissue.

No severe acute toxicity was observed. A mild skin reaction and mucositis occurred in 3 patients and temporary hair loss in 2 as acute toxicity. Late toxicity was observed in 2 of the 13 patients who were followed up for more than 6 months. One of these patients was a neonatal case with stage 3 disease who reached adulthood. Retrospective measurement of the mitosis-karyorrhexis index (MKI) of pathological samples showed a favorable histology consistent with International Neuroblastoma Pathology Committee (INPC) criteria (low MKI and ≤1 year old at diagnosis). The MYCN status was not measured, but the patient may have been classified into the intermediate risk group with non-amplified MYCN, based on her survival. Thus, for a similar contemporary case, radiotherapy and chemotherapy would have been reduced; however, pathological and biological data for INRG risk grouping were not available in 1982 and she received PBT of 28.6 GyE in 13 fractions to the upper abdomen at age 1 year old. She visited our hospital due to unexplained occasional stomach pain during early pregnancy at 28 years after the PBT. The pain increased after eating, which caused loss of appetite. Thus, the patient did not gain enough weight during pregnancy and this resulted in a premature birth. A CT scan after birth showed vertebral growth retardation and a narrowed aorta, including the celiac artery (Figure 4). The reason for the stomach pain was concluded to be mesenteric ischemia due to stenosis of the superior mesenteric artery. Cilostazol was prescribed to increase blood flow and her stomach pain was relieved. There were no symptoms caused by vertebral growth retardation. The other patient received 19.8 GyE to the skull when he was 2 year old (Figure 5). Hair loss was prolonged, and his hair has been thin with some white hair from 18 months after PBT. No secondary cancer was observed.

## Discussion

Recent progress of systemic therapies for advanced neuroblastoma has resulted in improved clinical outcomes, but local control is still an important concern. Panandiker et al. suggested that locoregional tumor control has an influence on overall survival [18], but gross resection is sometimes difficult because of bulky and invasive features involving critical organs. The prognosis of neuroblastoma with gross residual disease is poor and control is difficult, with Kushner et al. finding local recurrence in 3 of 7 patients with residual disease [3]. Neuroblastoma is highly sensitive to radiotherapy and a relatively low radiation dose of approximately 20 Gy is commonly used for minimal residual disease; however, the necessity for an escalated dose for gross residual disease has been suggested [19,20]. Kogan et al. reported an estimated 5-year locoregional recurrence rate of 51% for high risk primary neuroblastoma treated with 10 Gy for residual disease [19]. Compared with this, local recurrence was not observed in the patients with primary disease in our study, even though most patients had gross residual disease that was considered to be difficult to control by photon radiotherapy.

Recurrent neuroblastoma is also difficult to control, and sometimes acquires resistance to chemotherapy. Local radiotherapy for recurrent neuroblastoma has not been established. In our cases, most recurrent tumors were too large to be treated by photon radiotherapy, but around 20 Gy seemed to be insufficient for these tumors. Therefore, we treated 6 patients with recurrent disease using PBT with escalated doses from 30.6 to 45.5 GyE. Most of the patients eventually died, but 5 of the 6 had had an immediate complete response, with 2 surviving for more than 2 years after PBT and 1 patient still alive with no evidence of recurrence. These results suggest that PBT may contribute to improvement of the prognosis of patients with recurrent disease.

PBT is considered to be superior to intensity modulated radiation therapy (IMRT) for pediatric patients because higher doses can be delivered homogeneously to a large volume of neuroblastoma with a small number of ports while delivering very low doses in the path of the beam, which minimize the risk of a secondary cancer due to peripheral doses [10,12]. Hillbrand et al. suggested that PBT for pelvic neuroblastoma was preferred over IMRT because the dose distribution in IMRT produced a 1.5-fold greater risk of adverse events compared to PBT [10]. However, it is still important to monitor possible toxicity after PBT and we experienced late toxicities in 2 patients. Follow-up is particularly important for girls for whom the aorta was included in the treatment field in PBT and photon radiotherapy because this may affect a future pregnancy. The threshold of aortic retardation is unclear; therefore, irradiation of the aorta should be avoided as much as possible by taking advantage of the proton dose distribution. Thus, the indication for PBT and photon radiotherapy in infancy should be carefully considered. Hair loss is generally only a minor problem after photon radiotherapy at 20 Gy, but this effect was prolonged in one of our patients. This may be because proton beams do not have a build-up effect and doses at the skin surface are higher than those in photon radiotherapy when the target is located near the surface. We choose proton as an alternative to electron beams to reduce cranial doses, but hair follicles are located 4 mm deep in the skin and the lethal dose for hair follicles is 16 Gy [21]. This indicates that the treatment field and indication should be carefully determined to optimize the utilization of the characteristics of proton beams.

Until recently, PBT was viewed as a novel therapy, and therefore we had mainly used PBT to treat patients with neuroblastoma that was difficult to manage, with most of these patients having gross residual disease. Management with photon radiotherapy was difficult in 8 of the 14 patients in the study and PBT was chosen as a potential treatment option. The outcomes in these cases were favorable and little severe toxicity occurred, even though higher doses (> 20 Gy) were used for some patients. Our study is limited by the small number of patients and restriction of long-term follow-up to only one patient, but the results suggested that PBT may be conducted safely for patients with neuroblastoma that is difficult to manage using photon beams.

## Conclusions

PBT may be conducted safely for patients with neuroblastoma that is difficult to manage using photon beams.

## Abbreviations

PBT: Proton beam therapy; INSS: International Neuroblastoma Staging System; INRG: The international neuroblastoma risk group; IORT: Intraoperative radiotherapy; CTV: Clinical target volume; PTV: Planning target volume; MKI: Mitosis-karyorrhexis index; INPC: International Neuroblastoma Pathology Committee; IMRT: Intensity modulated radiation therapy.

## Competing interests

The authors declared that they have no competing interests.

## Authors’ contributions

YO selected the data and performed the analysis, drafted and wrote the manuscript. TF and TN planned and conducted chemotherapy. HO and MK performed surgery and chemotherapy. YO, MM, TO, SS, HI, TH, KT, and HS conducted radiotherapy. YO, TO, TF, MK, and HS reviewed/ revised the article. All authors read and approved final manuscript.
